# Exploration of the quantitative-effectiveness association between acupuncture temporal parameters and chemotherapy-induced peripheral neuropathy in cancer patients: a dose-response meta-analysis of randomized controlled trials

**DOI:** 10.3389/fonc.2024.1527331

**Published:** 2025-02-12

**Authors:** Hao Tian, Qin Luo, Liuyang Huang, Guang Chen, Mingsheng Sun, Fanrong Liang

**Affiliations:** ^1^ College of Acupuncture and Tuina/The 3rd Teaching Hospital/College of Basic Medicine/College of International Education, Chengdu University of Traditional Chinese Medicine/Clinical Research Center for Acupuncture and Moxibustion in Sichuan Province, Chengdu, Sichuan, China; ^2^ Department of Rheumatology and Orthopedics, Sichuan Province Orthopedic Hospital, Chengdu, Sichuan, China; ^3^ College of Acupuncture and Orthopedics, Hubei University of Chinese Medicine, Wuhan, China; ^4^ Hubei Provincial Collaborative Innovation Center of Preventive Treatment by Acupuncture and Moxibustion, Hubei University of Chinese Medicine, Wuhan, China; ^5^ Hubei Shizhen Laboratory, Hubei University of Chinese Medicine, Wuhan, China

**Keywords:** acupuncture, chemotherapy-induced peripheral neuropathy, pain management, meta-analysis, dose-response

## Abstract

**Background:**

Chemotherapy-induced peripheral neuropathy (CIPN) is one of the commonly reported symptoms impacting cancer survivors. This study evaluated and compared the effectiveness of acupuncture treatments for CIPN.

**Methods:**

We searched six databases from their inception to August 2024 to identify eligible randomized controlled trials (RCTs). Primary outcome were pain scores. Secondary outcomes were quality of life including FACT/GOG-Ntx and EORTC QLQ-C30. The robust error meta-regression (REMR) method was used to evaluate the dose-response relationship across treatment parameters, including number of sessions, frequency, and duration.

**Results:**

In total, 11 RCTs featuring 740 participants were included. The meta-analysis demonstrated that the primary analysis achieved a significant reduction in pain scores, with a standardized mean difference of [SMD= -1.23, 95% CI = (-2.22, -0.24); *P* < 0.01; *I²* = 95%], improvement quality of life including FACT/GOG-Ntx [SMD = 0.95, 95% CI = (0.02, 1.88); *P* < 0.01; *I²* = 93%] and EORTC QLQ-C30 [SMD = 0.36, 95% CI = (0.03, 0.68); *P* = 0.14; *I²* = 46%]. The nonlinear dose-response analysis suggests that pain improvement achieves the MCID at 16 treatment sessions, over 8 weeks, with a frequency of twice per week. Furthermore, analysis of the treatment duration chart shows that acupuncture maintains therapeutic effects during the follow-up period. Sensitivity analysis confirmed the robustness of these findings.

**Conclusion:**

Acupuncture demonstrates significant potential in managing CIPN, particularly through individualized treatment regimens. The identified time-dose-response relationship suggests that tailoring acupuncture frequency and duration can to optimize pain relief in CIPN patients. Future high-quality studies and large-scale multicenter clinical trials are needed to validate these findings.

## Introduction

Cancer is a leading global cause of mortality. According to estimates, nearly 20 million new cancer cases emerged in 2022, with projections indicating 9.7 million cancer-related deaths worldwide by 2030 (https://www.cancer.gov/about-cancer/understanding/statistics; last accessed on August, 2024). Chemotherapy-induced peripheral neuropathy (CIPN) is a prevalent adverse effect in cancer patients undergoing and post-chemotherapy, with approximately 30% to 60% of patients developing CIPN following neurotoxic chemotherapy (1). CIPN is characterized by structural and functional impairments of peripheral motor, sensory, and autonomic neurons (2, 3). The symptoms of CIPN vary based on the chemotherapy agent, with platinum-based drugs such as cisplatin, oxaliplatin, and carboplatin often leading to peripheral sensory neuropathy, and vinca alkaloids like vincristine or taxanes like paclitaxel causing mixed sensory and motor peripheral neuropathy, potentially with autonomic involvement (4, 5). CIPN arises when neurotoxic antineoplastic drugs accumulate and induce conduction dysfunction in the peripheral nervous system. Common neuropathic descriptions include numbness, tingling, and pain, typically presenting in a “stocking-glove” distribution, starting distally in the extremities and potentially progressing proximally as the condition deteriorates. The onset of CIPN symptoms often necessitates a reduction or cessation of chemotherapy, which can negatively impact tumor prognosis (6). According to the current American Society of Clinical Oncology (ASCO) guidelines, there are no recommended agents for the prevention of CIPN. Duloxetine stands as the sole agent with appreciate evidence to support its use in managing established painful CIPN in patients (6).

Acupuncture, a traditional medical therapy, involves the insertion of thin metal needles into specific anatomical points to stimulate the central and peripheral nervous systems. Some systematic reviews (7–9) have confirmed the efficacy of acupuncture in alleviating CIPN pain and improving quality of life. However, the specific impact of treatment frequency and duration on CIPN outcomes remains unexplored.

This study aims to use meta-analysis to evaluate the effectiveness of acupuncture in alleviating the pain symptoms and improving the quality of life in CIPN patients. Employing a robust error meta-regression model (REMR), it also explores the dose-response relationship between acupuncture temporal parameters—such as the number of sessions, frequency, and duration—and clinical efficacy in cancer patients with CIPN. The goal is to generate high-quality evidence to establish the effectiveness of acupuncture for CIPN treatment and to determine the optimal timing regimen.

## Methods

### Protocol and registration

This meta-analysis was registered on the PROSPERO platform (number: CRD42022357209) and reported following the Preferred Reporting Items for Systematic Reviews and Meta-Analysis checklist (10). See in **Supplementary 1**.

### Eligibility criteria and exclusion criteria

#### Types of studies

All English-language RCTs were considered eligible for inclusion, irrespective of geographical or publication status. In the case of randomized cross-over trials, only the first treatment period was included in the analysis. Exclusion criteria were applied to non-randomized clinical studies, quasi-RCTs, cluster RCTs, case reports, and studies where data were unavailable.

#### Types of participants

Eligible trials included those enrolling adults diagnosed with any form of cancer who were undergoing chemotherapy with drugs known for peripheral neurotoxicity and exhibited symptoms indicative of peripheral nerve injury.

#### Types of interventions

Acupuncture therapies were considered, including manual acupuncture (MA) plus usual care (UC), electroacupuncture (EA) plus UC were included.

#### Types of comparators

The control interventions comprised conventional treatment, usual care (UC), sham acupuncture (SA), and wait-list (WL) groups.

#### Types of outcome measurements

Studies were included if they measured at least one relevant outcome. The primary outcome in our meta-analysis were the pain scores, which were assessed using tools such as the Brief Pain Inventory (BPI), Visual Analog Scale (VAS), Neuropathic Pain Scale (NPS) for pain, specifically targeting neuralgia (11). Secondary outcomes, which indicate an improved quality of life, included the Functional Assessment of Cancer Therapy/Gynecologic Oncology Group-Neurotoxicity (FACT/GOG-Ntx) and current EORTC recommendations (12, 13). Outcomes were calculated as the absolute difference between post-treatment and pre-treatment values (mean ± standard deviation).

### Search strategy

#### Searching strategies

We systematically searched six databases—PubMed, Embase, Web of Science, and the Cochrane Central Register of Controlled Trials, WHO ICTRP, ChiCTR—from inception to August 2024 for relevant RCTs. The search was conducted using keywords such as ‘chemotherapy-induced peripheral neuropathy’, ‘CIPN’, ‘acupuncture’, ‘manual acupuncture’, ‘electroacupuncture’, ‘electro’, ‘randomized controlled trials’, and ‘RCT’. Two reviewers independently screened the literature by examining titles and abstracts, followed by a full-text review of potentially eligible studies to determine inclusion in the meta-analysis. See in **Supplementary 2**.

#### Study selection and data extraction

Data extraction was conducted independently by two reviewers, with disagreements being resolved through discussion. Titles, abstracts, and keywords were independently screened by TH and QL to identify duplicate trials and to exclude clearly ineligible studies. The full texts of the studies were then examined to confirm adherence to the inclusion criteria. Any disagreements on study eligibility were adjudicated by a third reviewer.

#### Quality assessment and data analysis

The risk of bias of methodological quality was assessed using the Cochrane Risk of Bias (ROB) Tool 2 (14). This tool comprised seven parts (randomization process, deviation from intended interventions, missing outcome data, measurement of the outcome, selection of the reported result, overall biases) and ranked the methodological quality as unclear, low, or high. A third party (MSS or FRL) was consulted to assist in the final decision-making process.

#### Statistical analysis

In this study, we utilized the REMR method to evaluate REMR analysis to investigate the potential effects of various time factors. We observed a nonlinear relationship between the number of sessions, duration, frequency, and outcomes. This phenomenon may be attributed to different analytical models. In clinical practice, the efficacy of acupuncture is closely related to the accumulation of “dose”, and the dose-response relationship does not follow a simple linear pattern. Therefore, a nonlinear model may be more appropriate to describe this relationship (15, 16). Employing Stata 18.0 software, we incorporated random effects to account for heterogeneity across studies. We standardized acupuncture treatment parameters, such as the number of sessions, frequency, and duration, along with outcome measures pain scores, to ensure comparability across studies.

To identify potential dose-response relationships and account for variability across studies, we applied a random-effects model with a significance threshold of *P* < 0.05. We addressed potential non-linear relationships by employing a restricted cubic spline model with two knots positioned at the 25th and 75th percentiles of the dose distribution (17). The model fit was evaluated using the Akaike Information Criterion (AIC) and the Bayesian Information Criterion (BIC), and the reasonableness of the data representation was confirmed through visual inspection of the dose-response curves (17, 18). These steps established a comprehensive framework for analyzing the dose-response relationship between acupuncture treatment parameters and outcomes in CIPN patients

For the assessment and visualization of the risk of bias, we utilized R 4.3.1 software along with the “robvis” package. Meta-analysis and subgroup analysis were performed using the ‘meta’ package, which employs a random-effects model (DerSimonian-Laird method) and *I²* statistics to quantify heterogeneity. The ‘forestplot’ package was used to generate forest plots. Sensitivity analysis, defined by *I²* ≥ 50% or Cochran’s Q test *p* < 0.05, was conducted using a leave-one-out method to assess the influence of each study on the overall effect size and heterogeneity (19).

In the analysis of treatment efficacy, effect sizes were determined by computing the differences in mean values and standard deviations between the intervention and control groups. These effect sizes were interpreted according to the following criteria: small effect size (0.2), moderate effect size (0.5), large effect size (0.8), and very large effect size (1.2). Hedges’s g was used for continuous outcomes, especially in studies with smaller sample sizes, with the 95% confidence interval (CI) determining statistical significance. Heterogeneity was assessed using Cochran’s Q test (*p*<0.05) and *I²* statistic, categorized as not be important; (<40%), moderate (30%–60%), substantial (50%–90%), or considerable (>75%). Where meta-analysis was infeasible, a narrative synthesis was conducted (19).

## Results

### Selection of eligible studies

**Figure 1:**
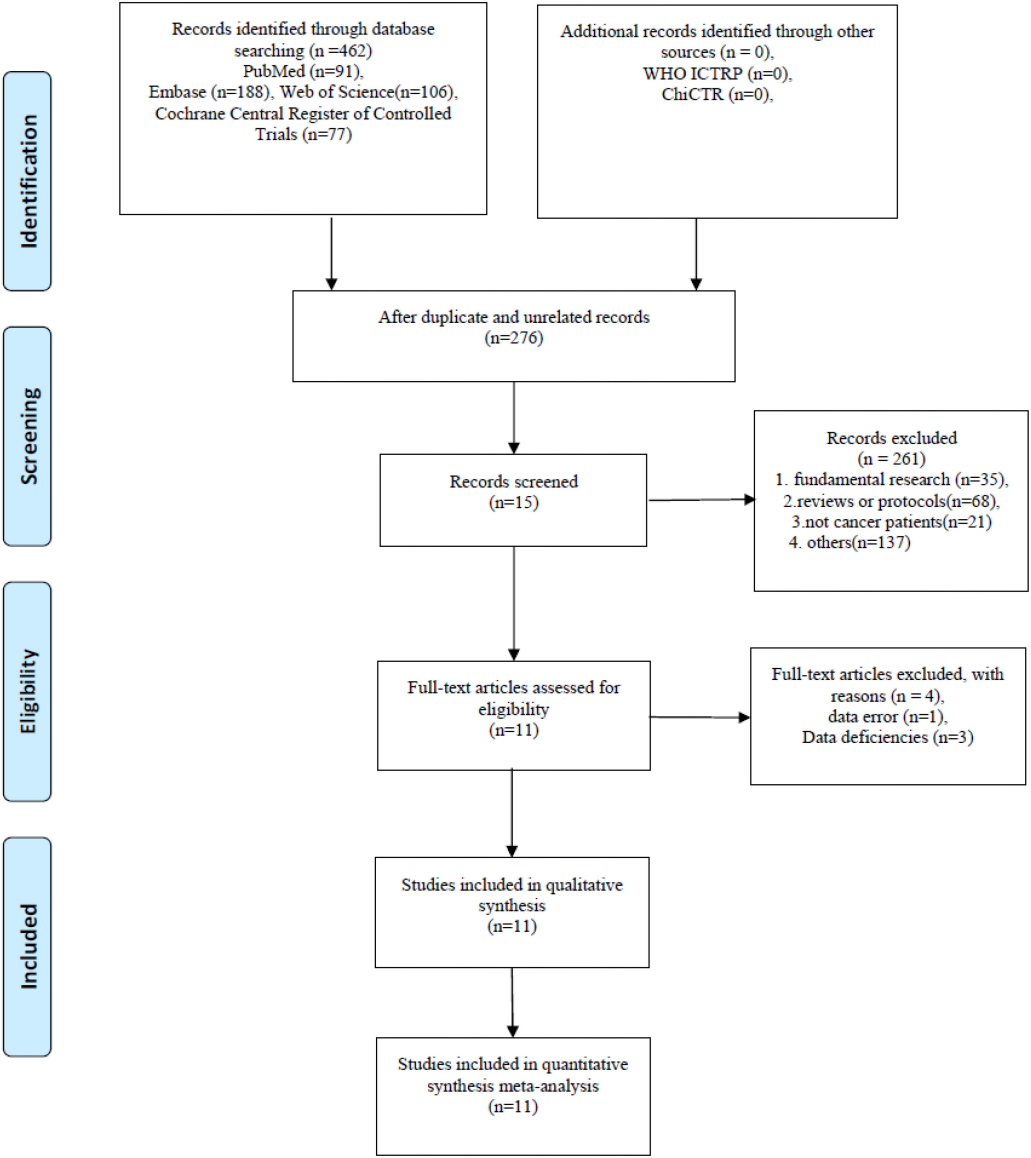
The PRISMA flow chart of selection process.

After the primary search process, we identified 462 potentially relevant studies from these databases. After eliminating 276 duplicates, we further excluded 35 fundamental research articles, 68 reviews or protocols, 21 articles that did not involve cancer patients, and other irrelevant studies. As a result, 15 articles were retained. Following a full-text assessment, 4 articles were excluded due to data errors and data deficiency. Ultimately, 11 RCTs were included in this systematic review **Figure 1**.

### Characteristics of the included studies

All included articles were in English, and **Table 1** presents the basic characteristics of the 11 studies included in the meta-analysis. The articles comprised of four for three-arm trials (20–23), one of four-arm trial (24), and the remaining six were two-arm trials (25–30). Four studies were conducted in the USA (21, 22, 26, 28), three in China (20, 27, 29), one in the UK (30), one in Brazil (25), one in Germany (24), and one in Israel (23). Sample sizes ranged from 15 to 69 participants, with an average sample size of 57, and participant ages ranged from 41 to 64. All studies provided detailed information on acupuncture points. The interventions included manual acupuncture (20–23, 25, 27–30) and electroacupuncture (24, 26), while control groups consisted of usual care (20, 21, 23–25, 27, 29, 30), and sham acupuncture (22, 26). Retention time for acupuncture generally ranged from 15 to 30 minutes. Acupuncture frequency varied: three studies applied it once per week (26, 28, 30), one at 1.5 times per week (18 sessions over 12 weeks) (27), two studies at 1.25 times per week (10 sessions over 8 weeks) (21, 22), three at twice per week (23, 25, 29), and two studies applied it three times per week (20, 24). Treatment duration ranged from 3 to 12 weeks, with a maximum follow-up time of 20 weeks (29). The total number of acupuncture sessions ranged from 8 to 18.

**Table 1:** Main characteristics of included RCTs.

Study	Patients	Country	Type of cancer	Age: mean	Experiment/Control	retention time	Frequency (per week)	Acupoints	Number of sessions (times)	Treatment duration (week)	Recording time points	Adverse events	Outcomes
Lu 2020 (28)	20/20	USA	Breast cancer	54.0 ± 41.8/53.5 ± 51.3	G1:MA G2: WL	30	Once a week	Yin Tang, LI11, TW5, Baxie, SP9, ST36, SP6, K3, LR3	8	8	0,4,8,12,16	Hematoma occurrence was 0.67%	PNQ, FACT-NTX, BPI-SF
Jacqui 2022 (30)	20/20	UK	Breast cancer, bone marrow cancer, gastrointestinal cancer, and gynecological cancer.	54(32.0 ± 68.0)/53.5 (26.0 ± 71.0)	G1:MA G2: UC	30min	Once a week	LV3, ST36, EXLE, BL60, LI4	10	10	0,10	16 adverse events (tingling, ache/pain, bruising, spotting of blood).	EORTC, QLQ-CIPN20, QLQ-C30
Eduardo 2018 (25)	15/14	Brasil	Cancer	41-82(57.68)	G1:MA G2: UC	30min	Twice a week	LR3, SP3, KI3, HT7, PC7, LU9	10	5	0,5	No adverse	NCI CTCAE/EORTC QLQ-C30/FIM/VAS
Alexander 2019 (29)	44/43	China, Hong Kong	breast, head and neck, colorectal, multiple myeloma, or gynecological cancer	/	G1:MA G2: UC	30min	Twice a week	LI4, LI11, PC7, TE5, Ex-UE9, SP6, ST36, LV3, ST41, and Ex-LE10.	16	8	0,8,14,20	No adverse	BPI/FACT/FACT GOG-Ntx/FACT-G/NCI-CTCAE (sensory, motor)
Bao 2020 (22)	24/23/21	USA	Patients with tumors	59.7(36. 3 ± 85.9)	G1:MA G2:SA G3: UC	20min	1.25 times per week	IL4, PC6, SI3, LR3, GB42, ST40, Bafeng 2, 3	10	8	0, 8	Adverse events were few and mild.	NRS (pain, tingling numbness)
Bao 2021 (21)	23/23/21	USA	Patients with tumors	59.7(36.3-85.9)/ 60.3(51.0-79.7)/ 62.7(43.0-86.0)	G1: electro G2: SA G3: UC	30min	1.25 times per week	Bilateral body: LI4, PC6, SI3, LR3, GB43, ST40, Bafeng 2, 3	10	8	0,4,8,12	RA group: 5 adverse events (e.g., needling site pain, bruising, claustrophobia with eye mask); SA group: 0 adverse events.	FACT/GOG-Ntx/HADS anxiety/HADS depression/ISI/BFI
Han 2017 (27)	49/49	China	Multiple myeloma	62.46/65.29	G1: MA+MET G2: MET	30min	1.5 weeks	In the supine position, the selected acupoints were bilateral LR3, ST43, GB41, SP6, ST36, SP10, and ST25; in the prone position, the acupoints included GV14, GV12, GV11, GV9, BL13, BL17, and BL58	18	12	0, 12	Five adverse events were reported (5:0)	BPI-SF/FACT-NTX/Neuropathic pain scale/VAS
Greenlee 2016 (26)	31/32	USA	Breast cancer	50 ± 11	G1:EA G2:SA	30	Once a week	The selected acupoints included GB34, ST36, LI4, LI10, Huatuojiaji at L3 and L5, Bafeng points on the feet, and Baxie points on the hands.	12	12	6,12,16	A single adverse event was reported: a grade 1 needle site reaction with discomfort, minor swelling, and bruising post-needle withdrawal.	BFI/FACT-NTX score/FACT-TAX total score/Neuropathic pain scale
Iravani 2020 (20)	19/19	China	Various types of cancer	64(46-79)	G1: MA G2:Vit B1 (21, 22)/gabapentin	20min	Three times per week	The selected general acupoints included CV6, GV20, bilateral ST36, SP6, LI4, LI11, and LR3, with additional points being bilateral Bafeng (EX-LE10) and Baxie (EX-UE9).	12	4	2,4,8	No adverse	NRS/CIPN/NCI-CTCAE/NCS/Electroneurographic
Rostock 2013 (24)	14/15/17	Germany	Various types of cancer	49.9 ± 9.6/ 52.3 ± 11.3 56.3 ± 11.1/ 52.0 ± 8.1	G1:EA G2: HB G3: VitB G4: placebo	15min	Three times per week	The selected acupoints included LV3, SP9, GB41, GB34, LI4, LI11, SI3, and HT3.	8 ± 1	3	7,12	/	CIPN score/Electroneurographic tests
Eran 2022 (23)	69/32	Israel	Breast Cancer	57.90 ± 12.1/59.90 ± 10.9	G1:MA G2:MA+CIM G3: UC	30min	Twice a week	The selected acupoints included LV3, SP9, GB41, GB34, LI4, LI11, SI3, and HT3, LR3, LR8, SP4, SP6, ST36, GB34, K3, and Bafeng.	12	6	0,6	No adverse events	FACT-Tax/EORTC QLQ-C30/Pain scale/QOL scale

CIPN, Chemotherapy-Induced Peripheral Neuropathy; EORTC QLQ-C30, European Organisation for Research and Treatment of Cancer Quality of Life Questionnaire – Core 30; NRS, Numerical Rating Scale; NCI-CTCAE, Common Terminology Criteria for Adverse Events; BPI, Brief Pain Inventory; FACT/GOG-Ntx, Functional Assessment of Cancer Therapy/Gynaecologic Oncology Group-Neurotoxicity; ISI, Insomnia Severity Index; HADS, Hospital Anxiety and Depression Scale; BFI, Brief Fatigue Inventory; CIM, Complementary and Integrative Medicine; MET, Methylcobalamin.

### Risk of bias among the included RCTs

**Figure 2:**
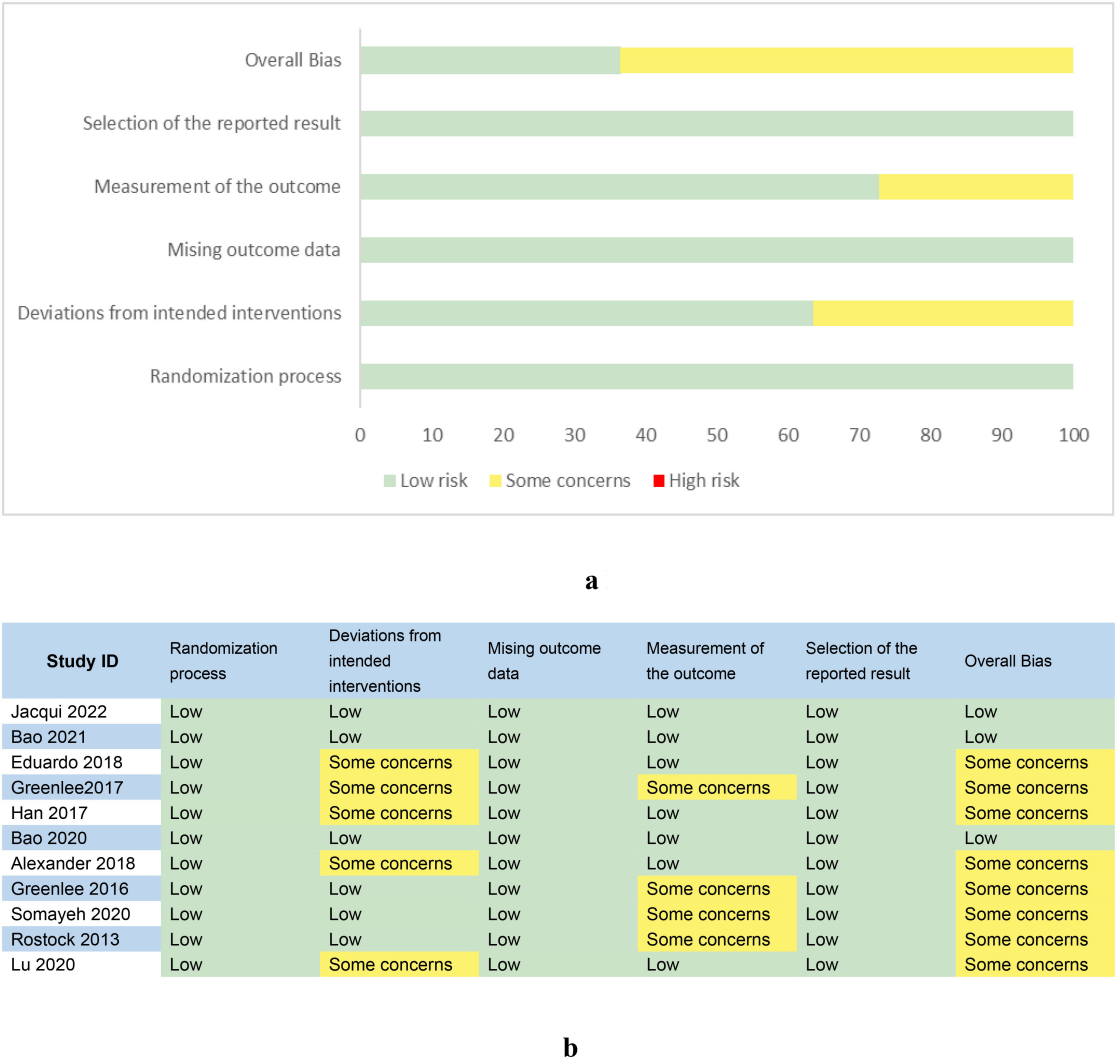
**(A)** Risk of bias graph. **(B)** Risk of bias summary.

The Cochrane ROB 2 Tool was used to assess the quality of all 11 trials included in our study. The overall risk of bias is presented in **Figures 2A, B**. To minimize bias risk, One RCT (27) reported using a random number table, while ten RCTs (20–26, 28–30) generated randomization lists by computer. In terms of allocation concealment, six trials (20–24, 30) reported allocation concealment and were considered to have a low risk of bias. Five trials did not describe allocation methods (25–29). Two trials (21, 22) described methods for blinding participants, four studies (23, 26, 29, 30) described blinding methods for acupuncturists, and seven studies (21–23, 25, 26, 28, 29) described blinding for outcome assessors. The remaining studies were considered to have an unclear risk of bias. All studies provided relevant protocols and were considered to have a low risk of bias for selective outcome reporting and other biases **Figures 2A, B**.

### Outcomes

#### Meta-analysis of the outcomes

Pain scores: Ten studies (20, 22–30) were evaluated in this manner, including 686 patients. The results indicated considerable heterogeneity among the studies, so a random effects model was used for statistical analysis. Acupuncture effectively alleviates pain in patients with CIPN. As shown in the figure, there was a significant difference between the experimental and control groups [SMD = -1.23, 95% CI = (-2.22, -0.24); *p <* 0.01; *I²* = 95%].

**Figure 3:**
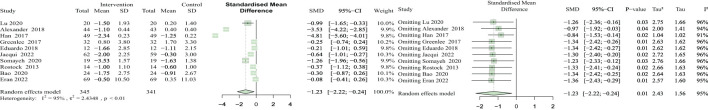
Forest plots for the effects of acupuncture on reducing pain and sensitivity analysis.

In the sensitivity analysis for the pain outcome, high heterogeneity was confirmed (*I²* = 95%). Sequential exclusion of individual studies showed minimal impact on the overall effect size and heterogeneity. Heterogeneity slightly decreased to I²= 91%, with the pooled effect size adjusted to SMD = -0.84 (95% CI: -1.53 to -0.14). Overall, the effect size remained statistically significant (*P* < 0.05) throughout the leave-one-out analysis, indicating the robustness of the findings (**Figure 3**).

**Figure 4:**
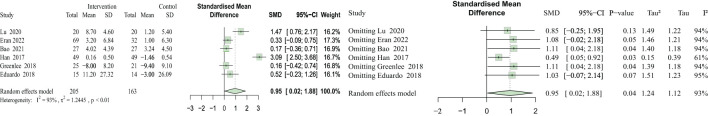
Forest plots for the effects of acupuncture on improvement FACT/GOG-Ntx and sensitivity analysis.

FACT/GOG-Ntx: A total of six studies (21, 23, 25–28), including 368 patients, were evaluated, and the results showed a beneficial effect of acupuncture compared to the control group [SMD = 0.95, 95% CI = (0.02, 1.88); *P <* 0.01] **Figure 4**.

In the sensitivity analysis for the FACT/GOG-Ntx outcome, high heterogeneity was confirmed (*I²* = 93%). Sequential exclusion of individual studies revealed that after removing one study with some quality concerns (27), reducing heterogeneity to *I²* = 61% and slightly altering the pooled effect size from SMD = 0.95, 95% CI = (0.02,1.88) to SMD = 0.49, 95% CI = (0.05,0.92). This suggests that while the exclusion of this study decreases heterogeneity, the overall effect size remains statistically significant, indicating the robustness of the findings.

**Figure 5:**
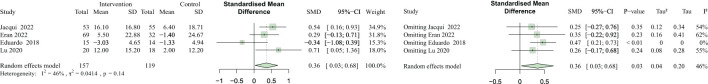
Forest plots for the effects of acupuncture on improvement EORTC QLQ-C30 and sensitivity analysis.

EORTC QLQ-C30 was reported in four studies (23, 25, 28, 30), including 276 patients. A random effects model analysis revealed that acupuncture significantly increased EORTC QLQ-C30 in CNPI patients compared to controls (SMD = 0.36, 95% CI = 0.03,0.68; *P =* 0.14; *I²* = 46%). Further sensitivity analysis demonstrated the robustness **Figure 5**.

In the sensitivity analysis for the EORTC QLQ-C30 outcomes, substantial heterogeneity was observed (*I²* = 95%). Sequentially excluding individual studies revealed that after removing one small-sample study (25), heterogeneity was notably reduced to *I²* = 0%. This adjustment led to a change in the pooled effect size from SMD = 0.36, 95% CI = (0.03, 0.68) to SMD = 0.47, 95% CI = (0.21, 0.73). These findings suggest that this particular study significantly contributes to the overall heterogeneity. However, even after its exclusion, the intervention’s effect on quality of life remains statistically significant, underscoring the robustness of the overall results.

#### Subgroup meta-analysis

This subgroup analysis focused on studies related to changes in pain and FACT/GOG-Ntx outcomes. The results indicate that, in terms of pain indicators, manual acupuncture (20–23, 25, 27–30) [SMD = -1.46, 95% CI = (-2.66,-0.27); *P* <0.01; *I²* = 96%] showed significant improvement compared to the control group. The duration of acupuncture treatment, whether it weeks>8 weeks (24, 27, 29, 30) [SMD =-2.33, 95% CI = (-4.46,-0.19); *P* <0.01; *I*² = 98%) or ≤ 8 weeks (20–23, 25, 26, 28) [SMD =-0.47, 95% CI = (-0.84, -0.09); *P* = 0.02; *I²* = 61%], both demonstrated statistical significance. In the control group, whether it was patients receiving only usual care (20, 23–25, 27, 29, 30) [SMD = -1.55, 95% CI = (-2.92,-0.18) *P* <0.01; *I²* = 97%] or those on a WL (28), [SMD = -0.99, 95% CI = (-1.65,-0.03)] the treatment group showed statistically differences when compared to them. Furthermore, studies with a larger sample size (more than 15 patients) (20, 22, 23, 26–30) [SMD = -1.46, 95% CI = (-2.65,-0.27); *I²* = 96%; *P* <0.01] also showed a statistically significant association with treatment efficacy. However, when the experimental group used EA [SMD = -0.29, 95% CI = (-0.70,0.13); *I²* = 95%; *P* = 0.79], or the control group used SA [SMD = -0.27, 95% CI = (-0.65,0.10); *I²* = 0; *P* = 0.89], or when the sample size was ≤15 [SMD = -0.30, 95% CI = (-0.84,0.25); *I²* = 0; *P* = 0.78], no statistically significant association was observed **Table 2**.

**Table 2:** Subgroup analysis of the effect on pain scores.

Outcomes	No. of studies	SMD	I^2^ (%)	*P* for heterogeneity	*P* for subgroup differences
Pain changes					
Overall	10	-1.23(-2.22, -0.24)	95%	*P* <0.01	*P =* 0.07
Acupuncture type					
Electro-acupuncture	2	-0.29(-0.70,0.13)	95%	*P* =0.79	
Manual acupuncture	8	-1.46(-2.66, -0.27)	96	*P* <0.01	
Control type					
WL	1	-0.99(-1.65, -0.33)	/	/	
Usual Care	7	-1.55(-2.92, -0.18)	97%	*P* <0.01	
SA	2	-0.27(-0.65,0.10)	0	*P* = 0.89	
Sample size					
>15	8	-1.46(-2.65, -0.27)	96%	*P* <0.01	
≤15	2	-0.30(-0.84,0.25)	0	*P* = 0.78	
Duration					
>8 week	4	-2.33(-4.46, -0.19)	98%	*P* <0.01	
≤8 week	6	-0.47(-0.84, -0.09)	61%	*P* = 0.02	
FACT/GOG-Ntx					
Overall	6	0.95(0.02,1.88)	93%	*P* <0.01	*P =* 0.48
>8 week	2	1.62(-1.25,4.49)	98%	*P* <0.01	
≤8 week	4	0.58(0.04,1.12)	68%	*P* = 0.03	

#### Dose-response meta-analysis

The study utilized the REMR method to identify the non-linear, quantitative-effectiveness relationship between acupuncture time parameters (treatment duration, frequency, and session) and pain outcomes in CIPN patients.

**Figure 6:**
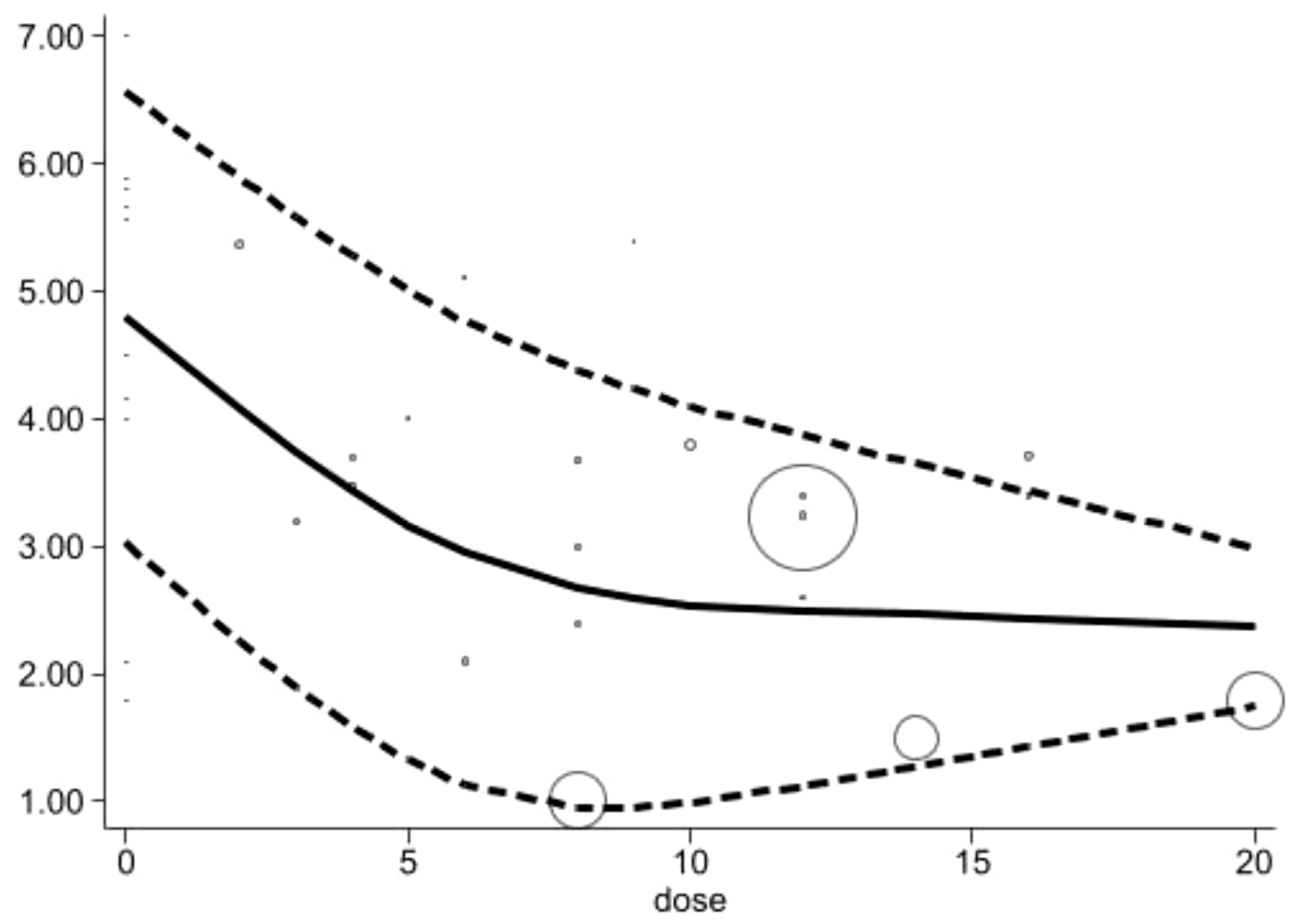
Dose-response relationships between acupuncture duration (week) and changes of pain scores.

Acupuncture duration (week): Within a certain range of treatment duration, there is a negative correlation between duration and pain levels, with pain scores continuously decline, forming an L-shape. At the baseline (0 weeks), the pain score was 4.80 (95% CI: 3.03–6.57). As the treatment duration increased, the pain score gradually decreased, with a substantial drop at 6 weeks to 2.95 (95% CI: 1.13–4.76). Subsequently, the reduction in pain score stabilized, ranging from 2.67 (95% CI: 0.95–4.38) at 8 weeks to 2.37 (95% CI: 1.75–2.99) at 20 weeks, indicating that the pain-relief effect reached a stable level **Figure 6**.

**Figure 7:**
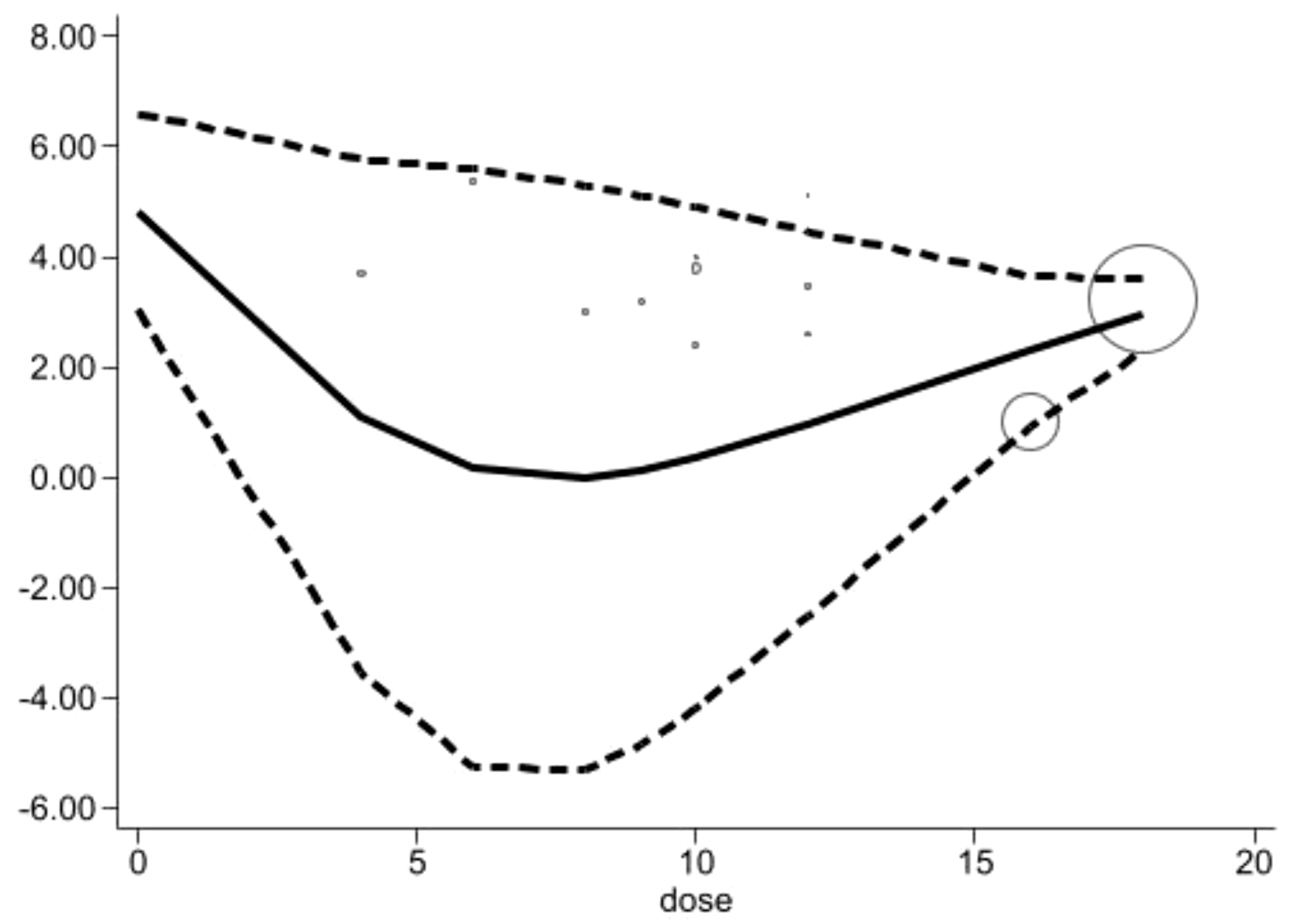
Dose-response relationships between the number of acupuncture sessions (times) and changes of pain scores.

Acupuncture session (times): Based on the data, as the number of acupuncture sessions increases, the pain score exhibits a V-shaped trend. Initially, the pain score is 4.82 (95% CI: 3.04–6.60). With an increase in sessions, the pain score gradually decreases, showing a noticeable drop at lower doses (between 4 and 8 sessions), reaching close to zero. However, within this session range from 4 sessions 1.11 (95% CI: -3.54–5.77) to 12 sessions: 0.97 (95% CI: -2.53–4.46). At higher doses (between 16 and 18 sessions), the pain score begins to rise, reaching 2.29 (95% CI: 0.93-3.64) and 2.95 (95% CI: 2.29-3.61), with confidence intervals entirely above zero. This indicates a statistically significant pain relief effect at these doses. Therefore, while lower to moderate doses of acupuncture show a decreasing trend in pain scores, this effect is not statistically significant; at higher doses, the pain relief effect stabilizes and becomes statistically significant **Figure 7**.

**Figure 8:**
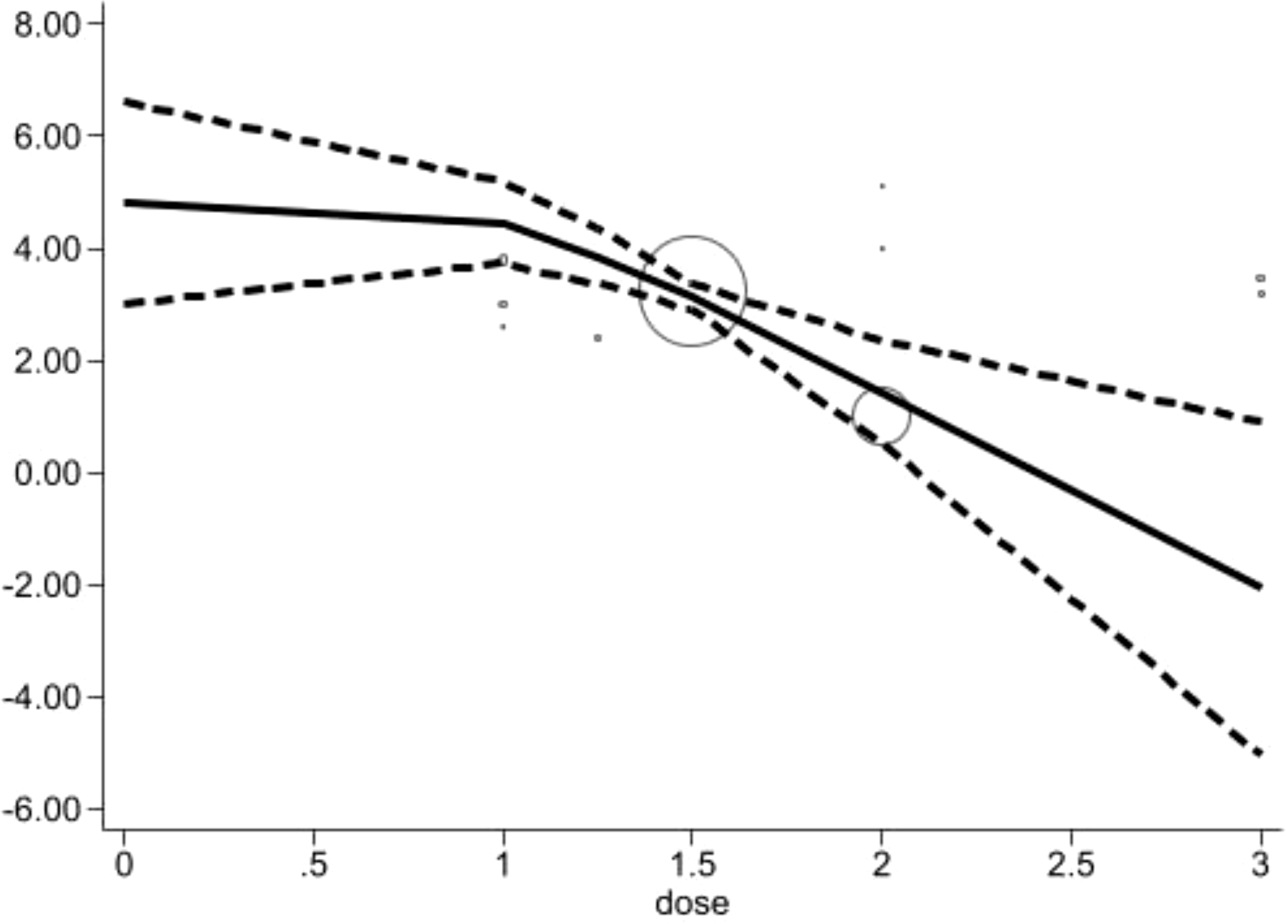
Dose-response relationships between acupuncture frequencies and changes of acupuncture frequencies (times per week).

Acupuncture frequencies (times per week): The chart shows a clear downward trend in pain scores as the acupuncture frequency increases which exhibits an inverted V-shaped trend. At the baseline, the pain score is relatively high at 4.81 (95% CI: 3.02-6.61). As the weekly frequency gradually increases to 1 and 1.5 times per week, the pain score steadily decreases to 4.46 (95% CI: 3.73-5.19) and 3.13 (95% CI: 2.90-3.36), indicating a negative correlation between frequency and pain level. At higher frequencies, such as 2 and 3, the pain score significantly drops to 1.44 (95% CI: 0.52-2.36). Subsequently, the pain score decreases further to -2.06 (95% CI: -5.03–0.90), suggesting substantial pain relief, despite the initial non-significant result **Figure 8**.

Integrating results. Based on the longitudinal analysis of time parameters, this study shows that acupuncture treatment duration exceeding 8 weeks, achieves the Minimum Clinically Important Difference (MCID). Within the first 6-8 weeks of treatment, the pain score shows a significant reduction, dropping to 2.95 (95% CI: 1.13-4.76) by the 6th week and further decreasing to MCID of 2.67 (95% CI: 0.95-4.38) at the 8th week, with minimal additional benefit from extending the treatment further. In addition, when the total number of sessions reaches 16-18, the pain relief effect becomes notable, with a score of 2.29 (95% CI: 0.93-3.64) at the 16th session, which is considered the optimal therapeutic dose, and then it slightly increases to 2.95 (95% CI: 2.29-3.61) at the 18th session. A frequency of twice times per week also significantly enhances pain relief, where the score drops to 1.44 (95% CI: 0.52-2.36). This treatment plan achieves optimal pain relief within a reasonable timeframe.

Adverse events: One trial (22) reported mild events, while other adverse reactions included discomfort, minor swelling, and bruising after acupuncture needle withdrawal (21, 26–28, 30). Four trials (20, 23, 25, 29) reported no adverse events, and one trial (24) did not mention any adverse events.

## Discussion

This study explores the efficacy of acupuncture for CIPN through a systematic review and meta-analysis. The development of peripheral neuropathy is a critical factor in limiting the dosage and duration of medication for cancer patients, as they often struggle to tolerate symptoms, leading to reduced doses, shortened treatment cycles, or even discontinuation of therapy (31). Therefore, alleviating CIPN during treatment is essential to improving the quality of life and treatment outcomes for patients undergoing chemotherapy.

Acupuncture, as a treatment for acute and chronic pain, is characterized by its low side effects. Chronic inflammation is a critical characteristic of malignant tumors, capable of inducing peripheral neuropathy and leading to neuropathic cancer pain. Acupuncture treatment has been proven to effectively alleviate peripheral nerve pain by regulating multiple systems, including the nervous, immune, and cardiovascular systems. The inflammatory tumor microenvironment, inflammatory reactions can promote the release of opioid peptides, which mediate analgesic effects through opioid receptors. Electroacupuncture treatment can induce peripheral tissue analgesia and hyperalgesia, which is associated with the involvement of β-endorphins, enkephalins, and dynorphins (32). Acupuncture may desensitize peripheral nerves by increasing the release of opioid peptides or activate immune cells to secrete opioid peptides, thereby raising local opioid peptide levels and exerting analgesic effects (33).Previous meta-analyses have shown that acupuncture significantly reduces pain scores and improves quality of life in CIPN patients (9, 34, 35). In particular, acupuncture has demonstrated improvements in FACT/GOG-Ntx and EORTC QLQ scores, enhancing both physical comfort and daily satisfaction for CIPN patients. This study further demonstrates that acupuncture, as a traditional non-pharmacological treatment, can significantly improve patients’ overall health status without increasing their medication burden. However, unlike previous studies, this research not only confirms the efficacy of acupuncture in CIPN pain management and quality of life improvement but also reveals a dose-response relationship between therapeutic effectiveness and treatment frequency and duration. Understanding this dose-response relationship provides a valuable foundation for developing personalized acupuncture treatment plans for CIPN. Subgroup analyses were conducted to explore potential influencing factors, including acupuncture type, control type, treatment duration, and sample size. Results showed that differences in efficacy across different acupuncture types (MA) and control types (WL and UC), as well as treatment durations divided at the 8-week threshold (with most studies using 8 weeks as the standard), were all statistically significant. Among them, MA proved more effective in pain relief. There is a lack of consensus regarding the benefits of MA and EA. Some studies suggest that EA, which uses electrical stimulation, may cause a stronger pricking or tingling sensation, thereby increasing patients’ pain sensitivity and providing better analgesic effects (36–38). In contrast, MA involves manual stimulation of acupoints, which is relatively gentler and may be more acceptable for some patients, potentially leading to greater pain relief (39). Among control types, acupuncture compared with the WL and UC groups still showed some improvement, whereas the SA group did not show a statistically significant difference from the experimental group. Additionally, studies with a sample size (>15) showed significant pain improvement, while those with a smaller sample size (≤15) displayed weaker effects that did not reach statistical significance. In summary, the subgroup analysis suggests that MA, larger sample sizes, certain treatment durations may be associated with greater efficacy in alleviating pain from CIPN, with larger sample sizes enhancing statistical significance. Although substantial heterogeneity was observed across studies, the subgroup analysis did not significantly reduce this heterogeneity; however, sensitivity analysis confirmed the robustness of the results.

This study is the first to explore the time-dose-response relationship between acupuncture and the improvement of pain levels in CIPN patients. Furthermore, it is the first to integrate and assess the “dose” of acupuncture across three-time parameters: number of sessions, frequency, and duration.

Studies have shown that in pain measurement tools such as the NPS, VAS and BPI, a change of approximately 2 points is generally considered the MCID, as it signifies notable pain relief for patients (40). In this study, acupuncture treatment helped CIPN patients reach the MCID level by the 8 weeks, and pain continues to decrease in subsequent treatments. The optimal acupuncture frequency is twice per week. The conclusion that aligns with the US Medicare Utilization Management Policy (UM Policy) for Determining Maximum Therapeutic Benefit (MTB). This policy assesses the maximum therapeutic benefit, including both pharmacological and non-pharmacological evidence, such as acupuncture. It shows that most patients experience significant pain or functional improvement within 2-6 weeks of treatment, with no additional benefits observed when extending treatment to 6-12 weeks. If there is no effect within the first 6 weeks, further treatment is unlikely to be effective (15).

Our findings align with this response curve, indicating that the relationship between the number of treatment sessions and efficacy is not linear but rather shows a “V-shaped” nonlinear pattern, with more than 16 sessions being critical for achieving optimal relief. Additionally, an “L-shaped” trend observed in treatment duration suggests that pain relief stabilizes after 8 weeks of acupuncture, with minimal additional benefit from extending the treatment further. The inverted “V-shaped” trend in weekly frequency indicates that a frequency of twice per week yields the best therapeutic outcomes. This dose-response relationship provides valuable guidance for developing individualized acupuncture treatment plans for CIPN patients.

### Strengths and limitations

This meta-analysis offers several key strengths. First, it is the first study to examine the time-dose-response relationship of acupuncture on pain outcomes in CIPN patients, providing a detailed evaluation of treatment parameters (frequency, duration, and number of sessions) that contribute to optimal pain relief. This in-depth analysis offers valuable insights for establishing individualized acupuncture treatment protocols. Second, the included studies are of high quality, and our analysis ensures broad representation across acupuncture techniques, various control groups, and different sample sizes, thereby enhancing the generalizability of the findings. Third, the robust methodological approach, including subgroup and sensitivity analyses, effectively addresses potential heterogeneity and confirms the stability of the results. Additionally, a nonlinear dose-response model was applied, accurately representing the complex relationship between treatment variables and outcomes rather than relying on simpler linear assumptions. This approach reveals the threshold at which acupuncture provides the most clinically significant benefits.

Despite its strengths, this study has several limitations. Firstly, many of the included trials had small sample sizes, with some involving fewer than 15 participants, which limits the statistical power and generalizability of the findings. Future studies should aim for larger sample sizes and involve multiple centers to enhance both statistical power and generalizability. Secondly, despite subgroup and sensitivity analyses, heterogeneity among the studies remained high (with *I²* values up to 95%), likely due to differences in acupuncture protocols, such as the selection of acupoints and needle retention times, as well as variations in patient populations. Standardizing acupuncture protocols, including the use of fixed acupoints and retention times, could help reduce heterogeneity and improve the reproducibility of the results. Additionally, some studies lacked detailed information on control group setup and blinding methods, which may introduce bias and further limit the generalizability of certain conclusions. Most included studies reported only baseline and outcome data, potentially impacting the robustness of the modeling and conclusions. Lastly, while this study primarily focused on MA and EA, the time-dose-response relationships of other acupuncture methods may differ and require additional exploration.

## Conclusion

In conclusion, this meta-analysis indicates that acupuncture has significant potential in reducing pain and improving quality of life for patients with CIPN. The study identifies a time-dose-response relationship, suggesting that pain relief can be achieved MCID with 16 treatment sessions, over 8 weeks, at a frequency of twice per week. This nonlinear relationship underscores the importance of individualized acupuncture regimens for CIPN, providing valuable guidance for clinical treatment protocols. However, due to limitations in study quality, further high-quality research and large-scale multicenter clinical trials are needed to confirm these findings and validate the efficacy of acupuncture.
