# Supplementary material for: Exploration of the quantitative-effectiveness association between acupuncture temporal parameters and chemotherapy-induced peripheral neuropathy in cancer patients: a dose-response meta-analysis of randomized controlled trials
Source: Front Oncol. 2025 Feb 12;14:1527331. doi: 10.3389/fonc.2024.1527331 (PMC11873836; doi:10.3389/fonc.2024.1527331)
Supplement: Supplementary file 2 [file DataSheet2.doc]

PubMed：

| #1 | ("Peripheral Nervous System Diseases"[MeSH Terms] OR "chemotherapy induced peripheral neuropathy"[Title/Abstract] OR "peripheral neuropathy"[Title/Abstract] OR "peripheral neurotoxicity"[Title/Abstract] OR "CIPN"[Title/Abstract] OR "chemotherapy induced neurotoxicity"[Title/Abstract] OR "neuralgia"[Title/Abstract] OR "paresthesia"[Title/Abstract] OR "neurotoxicity"[Title/Abstract] OR "neuropathy"[Title/Abstract]) | 284,883 |
| --- | --- | --- |
| #2 | ((Acupuncture Therapy[MeSH Terms] OR Acupuncture[MeSH Terms] OR Electroacupuncture[MeSH Terms]) OR (Acupuncture Therapy[Title/Abstract]) OR (Acupuncture[Title/Abstract]) OR (Electroacupuncture[Title/Abstract]) OR (Acupuncture Treatment[Title/Abstract]) OR (Acupuncture Treatments[Title/Abstract]) OR (Treatment, Acupuncture[Title/Abstract]) OR (Therapy, Acupuncture[Title/Abstract])) | 40,893 |
| #3 | #1 AND #2  (((Acupuncture Therapy[MeSH Terms] OR Acupuncture[MeSH Terms] OR Electroacupuncture[MeSH Terms]) OR (Acupuncture Therapy[Title/Abstract]) OR (Acupuncture[Title/Abstract]) OR (Electroacupuncture[Title/Abstract]) OR (Acupuncture Treatment[Title/Abstract]) OR (Acupuncture Treatments[Title/Abstract]) OR (Treatment, Acupuncture[Title/Abstract]) OR (Therapy, Acupuncture[Title/Abstract]))) AND (("Peripheral Nervous System Diseases"[MeSH Terms] OR "chemotherapy induced peripheral neuropathy"[Title/Abstract] OR "peripheral neuropathy"[Title/Abstract] OR "peripheral neurotoxicity"[Title/Abstract] OR "CIPN"[Title/Abstract] OR "chemotherapy induced neurotoxicity"[Title/Abstract] OR "neuralgia"[Title/Abstract] OR "paresthesia"[Title/Abstract] OR "neurotoxicity"[Title/Abstract] OR "neuropathy"[Title/Abstract])) | 1,468 |
| #4 | #1 AND #2 Filters: Clinical Trial, Randomized Controlled Trial  (((randomized controlled trial[pt] OR RCT[Title/Abstract] OR "randomized controlled trial"[Title/Abstract])) AND ((((Acupuncture Therapy[MeSH Terms] OR Acupuncture[MeSH Terms] OR Electroacupuncture[MeSH Terms]) OR (Acupuncture Therapy[Title/Abstract]) OR (Acupuncture[Title/Abstract]) OR (Electroacupuncture[Title/Abstract]) OR (Acupuncture Treatment[Title/Abstract]) OR (Acupuncture Treatments[Title/Abstract]) OR (Treatment, Acupuncture[Title/Abstract]) OR (Therapy, Acupuncture[Title/Abstract]))) AND (("Peripheral Nervous System Diseases"[MeSH Terms] OR "chemotherapy induced peripheral neuropathy"[Title/Abstract] OR "peripheral neuropathy"[Title/Abstract] OR "peripheral neurotoxicity"[Title/Abstract] OR "CIPN"[Title/Abstract] OR "chemotherapy induced neurotoxicity"[Title/Abstract] OR "neuralgia"[Title/Abstract] OR "paresthesia"[Title/Abstract] OR "neurotoxicity"[Title/Abstract] OR "neuropathy"[Title/Abstract])))) AND (("Peripheral Nervous System Diseases"[MeSH Terms] OR "chemotherapy induced peripheral neuropathy"[Title/Abstract] OR "peripheral neuropathy"[Title/Abstract] OR "peripheral neurotoxicity"[Title/Abstract] OR "CIPN"[Title/Abstract] OR "chemotherapy induced neurotoxicity"[Title/Abstract] OR "neuralgia"[Title/Abstract] OR "paresthesia"[Title/Abstract] OR "neurotoxicity"[Title/Abstract] OR "neuropathy"[Title/Abstract])) | 91 |
